# Comparative Genomics of Genes for rhamnolipid synthesis and monoaromatic hydrocarbon tolerance in environmental strains of *Pseudomonas aeruginosa*

**DOI:** 10.12688/f1000research.158761.1

**Published:** 2024-12-13

**Authors:** ROGER A. PALOMINO HUARCAYA, Camila Castillo-Vilcahuaman, Sandro B. Martel-Torres, Fernando A. Merino Rafael, Susana M. Gutiérrez Moreno

**Affiliations:** 1 Laboratorio de Microbiología y Biotecnología Microbiana, Universidad Nacional Mayor de San Marcos Facultad de Ciencias Biologicas, Lima District, Lima Region, +51, Peru; 2 Laboratorio de Genómica Microbiana, Universidad Peruana Cayetano Heredia, Lima District, Lima Region, +51, Peru

**Keywords:** Pseudomonas aeruginosa, BTEX, genes rhlABC, genes mlaABCD, genes pqs

## Abstract

**Background:**

Aromatic hydrocarbons such as Benzene, Toluene, Ethylbenzene and Xylene (BTEX), give us an environmental challenge. They can be degraded by strains of *Pseudomonas aeruginosa*, through emulsification ( *rhl* genes) and biodegradation processes. The BTEX tolerance genes ( *mlaABCD*) may be present in it. The aim of this research was to evaluate, through comparative genomics, the genes of the *rhl* and *mla* systems in six strains isolated from hydrocarbon polluted environments.

**Methods:**

This study examines six Pseudomonas aeruginosa strains isolated from hydrocarbon-contaminated sites in Peru. Each strain shows capabilities like hydrocarbon degradation and heavy metal tolerance. After DNA extraction, sequencing, and quality-controlled assembly, functional genome annotation was performed using BAKTA. Comparative analysis included high-quality *Pseudomonas* genomes from RefSeq, with ANI metrics to assess genetic similarity. A phylogenetic tree, built from core gene alignment, reveals evolutionary connections and was visualized with iTOL.

**Results:**

Results showed that all strains had a double copy of the *rhlB* gene, arranged in the same organization, with *rhlABRI* genes always adjacent in a 3′-5′ direction. However, *rhlG* varied in position and orientation among strains, often near *rhlC.* The C1BHIC5 strain had only one copy of the rhlB gene, and the 3′ – 5 orientation of the *rhlG* gene was the exception. Most strains had two copies of *mlaA*, *mlaFEDC*, and *mlaEFD* genes for toluene tolerance, with diverse orientations across strains. High synteny was noted within *mla* gene blocks. Compared to *Pseudomonas putida*, where *mla* genes are positioned between *murA* and *ppcD* with an additional toluene tolerance gene ( *ttg2D*), strains of *P. aeruginosa* strains display a similar yet distinct gene arrangement.

**Conclusions:**

In conclusion, the presence of the *rhlABC* genes in all the strains analyzed and the BTEX tolerance genes allowed us to understand the great ability of *P. aeruginosa* to survive in polluted environments.

## Introduction

Polycyclic aromatic hydrocarbons (PAHs) and BTEX aromatic hydrocarbons (benzene, toluene, ethylbenzene and xylene), widely distributed in the environment in complex mixtures, give us a significant environmental challenge due to their abundance and global recognition as pollutants ( Andreoni & Gianfreda, 2007). The World Health Organization has also recognized BTEX as potent carcinogens ( Chicca et al., 2020). Petroleum hydrocarbon spills are a serious environmental problem, as they constitute a source of soil and water pollution, disrupting aquatic and terrestrial ecosystems by influencing their structure and biological processes ( Shi et al., 2015). Because of their persistence in the environments, they cause harm to human health and to flora and fauna ( Dongen, 2000).

In spite of the toxicity risks, hydrocarbon polluted places are the best source to isolate aromatic and aliphatic hydrocarbon degrading microoganisms ( Salgado Brito et al., 2008). Among these microorganisms are bacteria belonging to various genera such as *Bacillus*, *Burkholderia*, *Acinetobacter*, *Rhodococcus*, and *Pseudomonas* ( Ghosal et al., 2016). For instance, strains like *Pseudomonas aeruginosa* N002, *P. aeruginosa* SJTD-1, and *P. aeruginosa* DQ8 have been identified as alkane-degrading organisms, while *Massilia aromaticivorans*, *Pseudomonas putida*, *Pseudomonas fluorescens*, *Rhodococcus*, *Bacillus* sp., and *Streptomyces* sp. have shown potential for BTEX degradation ( Kaur et al., 2023). Additionally, *Acinetobacter* spp., *Alcaligenes* spp., *Acidovorax* spp., *Janibacter* spp., and *Rhodococcus* spp. have been reported to be capable of degrading PAHs ( Shahsavari et al., 2019).

Studies related to the characterization and annotation of genes involved in hydrocarbon degradation are useful to understand the physiological and metabolic processes in the intrinsic dynamics of native microorganisms at the level of molecular biodiversity, genomics, transcriptomics, and metabolomics ( Kumari et al., 2023).

In *Pseudomonas aeruginosa*, it is necessary to consider the quorum sensing hierarchical complex network ( García-Reyes et al., 2020; Kuang et al., 2020; J. Lee & Zhang, 2015; Meng et al., 2020): the *rhl* system ( *rhlABR* operon, *rhlC*, *rhlI*, and *rhlG* genes); *las* system ( *lasR*, *lasI* genes) ( Kuang et al., 2020; Meng et al., 2020; O’Connor et al., 2022); PQS system ( *pqsABCBDE* operon, *pqsH*, *pqsL*, and *pqsR* or *mvfR* genes) ( Meng et al., 2020; O’Connor et al., 2022; Xiao et al., 2006); and the IQS system ( *ambBCDE* operon, *iqsR* gene) (J. Lee & Zhang, 2015; O’Connor et al., 2022). Additionally, genes responsible for BTEX tolerance ( *mlaABCD* genes), as well as those involved in alkane degradation ( *alkB* gene) ( Cruz et al., 2020), BTEX ( *tod*, *tom*, *tbu*, *tou*, *pTOL* pathways), and PAHs (TOD, NAH, OCT plasmid genes, among others), have been reported.

Studies of genes related to the degradation of monoaromatic hydrocarbons have been carried in *Pseudomonas putida.* The toluene-degrading plasmid (TOL) has been studied in *P. putida* mt-2, which encodes metabolic pathways for the degradation of toluene, m-xylene, and p-xylene into carboxylic acids. The *xylN* genes of the TOL plasmid encode a m- xylene transporter porin ( Kasai et al., 2001). Similarly, the degradation of toluene follows six differentiated metabolic pathways, which involve the *tod* gene encoding toluene dioxygenase, the *tmo*/ *tbm*/ *tbc* genes responsible for synthesizing toluene 2-monooxygenase enzyme, *tbu*/ *tbh* genes encoding toluene-3-monooxygenase, *tmoABCDEF* genes encoding different subunits of ortho toluene 4-monooxygenase protein, and *tbc1FEDCBA*/ *tbc2ABCDEF* genes encoding meta toluene 4-monooxygenase ( Parales et al., 2008). On the other hand, the genes involved in the aerobic degradation of benzene are the *benzA* genes encoding an enzyme that carries out the initial ring cleavage oxidation reaction of monoaromatic hydrocarbons, and this enzyme has been studied in *Pseudomonas putida* AQ8 ( Chicca et al., 2020). To date, there are not many records involving strains of the species *Pseudomonas aeruginosa* in the degradation of monoaromatic hydro- carbons, nor annotated genes associated with this process.

Research on hydrocarbon degradation has extensively explored various species of *Pseudomonas*, particularly focusing on their ability to break down complex organic compounds in diverse environments. *Pseudomonas aeruginosa*, renowned for its fluoranthene degradation capability, exhibits robust metabolic capacities. Investigations on this species have spanned multiple domains, including genomics, transcriptomics, metabolomics, and its potential applications in biotechnology ( He et al., 2018).

This research is focused in comparing the genetic characterization of genes coding for rhamnolipids production ( *rhl* ) and BTEX degradating genes in six *Pseudomonas aeruginosa* strains isolated in hydrocarbon polluted environments.

## Methods

### Selected strains

**Figure 1.**
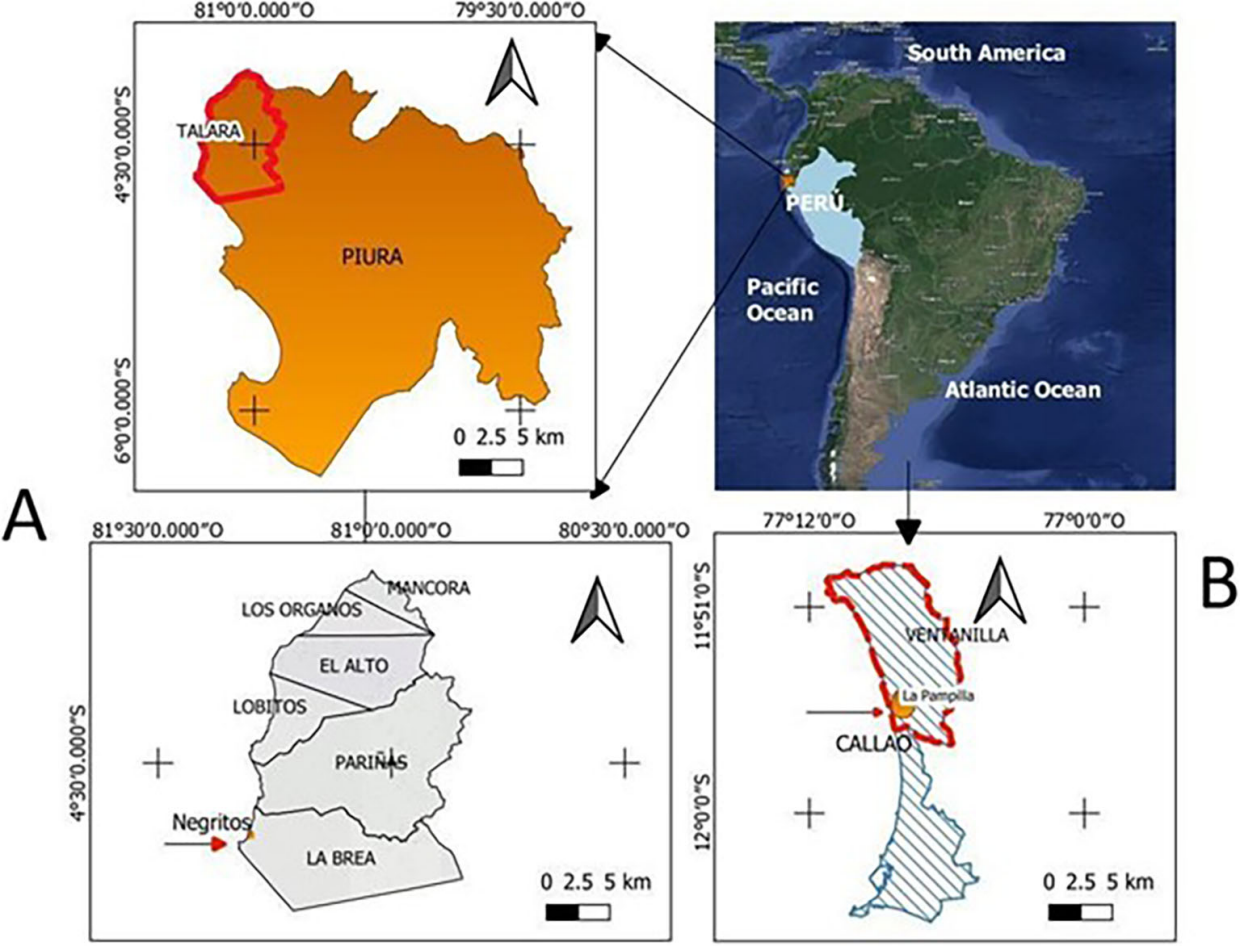
Geographic location of sampling sites: A) Talara Refinery (Northern Peru), B) La Pampilla (Callao-Lima). Elaboration: Figure created by the authors as part of this study.

Six strains of *Pseudomonas aeruginosa* isolated from hydrocarbon contaminated environments, from different geographical sites in Peru ( Figure 1) were selected ( Table 1); belonging to the collection of microorganisms of the Laboratory of Microbiology and Microbial Biotechnology (LAMYBIM) of the Faculty of Biological Sciences of the U.N.M.S.M. ( Palomino et al., 2017).

**Table 1.** Samples collected and names given to the strains.

Selected strains	Codes	Sampling location	Geographic location	Reference
Rhmanolipid hyperproducer	6K-11	Environments contaminated with hydrocarbons	Talara, Northern Peru	Tabuchi et al., 2015
With emulsifying capacity and heavy metal removal efficiency	PB25	Environments contaminated with hydrocarbons	La Pampilla, Ventanilla	Giraldo et al., 2014
With Hydrocarbonoclastic capacity	C1BHIC5	Land treatment field contaminated with crude oil	Talara, Northern Peru	Terán Morales, 2020
With Hydrocarbonoclastic capacity	C3ACETC53a	Land treatment field contaminated with crude oil	Talara, Northern Peru	Terán Morales, 2020
Non-rhamnolipid producer	2K-1	Environments contaminated with hydrocarbons	Talara, Northern Peru	Palomino et al., 2017
Non-rhamnolipid producer	3K-6	Environments contaminated with hydrocarbons	Talara, Northern Peru	Palomino et al., 2017

Elaboration: Table created by the authors as part of this study.

### Extraction of DNA and genome sequencing

For the purification process, the strains were subcultured in glass flasks containing 50 mL of Luria Bertani medium, at 37 °C overnight. Subsequently, the methodology of the innuPREP Bacteria DNA kit from analytikjena was followed.

Raw reads were verified with FastQC software ( Andrews, S., 2010) and filtered to remove contaminating Illumina adapter sequences and quality trimmed with Trimmomatic v0.36 ( Salgado Brito et al., 2008). The resulting filtered reads were assembled using SPAdes v3.14.1 ( Ghosal et al., 2016). Assemblies were then filtered to contain only contigs longer than 400 bp. The quality of the assemblies was checked with Quast ( Ivanova et al., 2022). For validation assembly, we used Busco ( Khan et al., 2001) and mapped reads filtered against the draft genome using Bowtie2 ( Zylstra & Gibson, 1989) and Samtools ( Furukawa et al., 1993). For annotation, we used the Prokka pipeline, which used Prodigal ( Harayama & Rekik, 1993), RNAmmer ( Reineke, 1998), Aragorn ( Mapelli et al., 2017), and MinCED ( Skennerton et al., 2021) to find protein-coding genes, RNA, tRNA, and CRISPR regions, respectively. Finally, we found groups of orthologs using Orthofinder ( Cao et al., 2015), adding the proteome of a *Pseudomonas aeruginosa* strain (GCA_000006765.1) for this analysis.

### De novo assembly and validation

Trimming quality (Phred Q > 25) and removal of adapters were conducted with Trimmomatic v0.36 ( Bolger et al., 2014) and TrimGalore software (Felix Krueger et al., 2023), respectively. De novo assembly was performed using SPAdes v3.10.1 ( Bankevich et al., 2012), testing different k-mers (from 23 to 123). SSPACE v2.0 ( Boetzer et al., 2011) was used to join contigs to build scaffolds with an iteration of raw read alignment over the contigs to minimize the gaps. A final gap-filling step was performed using GapCloser ( Boetzer & Pirovano, 2012) to generate a draft genome. We used QUAST v5.2.0 ( Gurevich et al., 2013) for assembly statistics. The completeness and consistency of the assembled genome were estimated using the Benchmarking Universal Single-Copy Orthologs (BUSCO) software ( Simão et al., 2015) and CheckM ( Parks et al., 2015). These assemblies have been deposited at DDBJ/ENA/GenBank under the accessions: JAOAQG000000000, JAOAQH000000000, JAOAQI000000000, JAOAQJ000000000, JANATH000000000, JANZYL000000000.

### Genomic functional annotation

The functional annotation of the obtained genomes was carried out using BAKTA v1.6.1 ( Schwengers et al., 2021), a precise annotation tool, using a local conda environment (Anaconda v23.5.0). We used BAKTA’s database in its 2022-08-25 release. Standard parameters were used for this functional annotation.

### Pseudomonas high quality genome sequence retrieval

For comparison purposes, *Pseudomonas* genomes were retrieved from public repositories based on their quality and completeness. All considered genomes of the *Pseudomonas aeruginosa* species were downloaded from the Reference Sequences Database for comparison (RefSeq; October 2022). Genomes were selected based on the quantity and quality according to the tool QUAST v5.2.0 ( Gurevich et al., 2013).

### Comparative genomic analysis and phylogenetic reconstruction

Average Nucleotide Identity (ANI) analysis was performed using pyANI (v. 0.2.12) ( Pritchard et al., 2016). A heatmap was generated using the pheatmap package in R (v. 4.1.2). A tblastx was performed using the assembled genomes with a focus in 2 genes of interest, and the genetic diagram was built using the genomes package in R. The phylogenetic reconstruction of multiple *Pseudomonas aeruginosa* strains was built, leveraging the core gene alignment obtained during pangenome reconstruction through Panaroo ( Tonkin-Hill et al., 2020). Subsequently, RAxML v8.2.13 ( Stamatakis, 2014) was employed to construct a phylogenetic tree based on the aligned core genes. The model was selected using ModelTest NG ( Darriba et al., 2020), with the TVM+I+G4 model chosen as the optimal one for this analysis. The phylogenetic analysis was run with 100 bootstraps. The phylogenetic tree was graphically edited using the iTOL v5 web tool ( Letunic & Bork, 2021).

For gene topology and synteny, we performed a tblastx between the contigs of interest. We performed this for 2 genes of interest: *rhl* and *mla.* For drawing the topology and the synteny obtained, we used the R package, gggenome ( Hackl et al., 2024). For the *rhl* system, we filtered the tblastx results based on E-value (<0.01), coverage length (≥20) and identity (≥30). For the *mla* system, we filtered by criteria based on E-value (<0.01), coverage length (≥100) and identity (≥30). PB25’s alignment against *P. putida* was filtered following more lax criteria (E-value (<0.01), coverage length (≥20) and identity (≥30)).

## Results

### Sequenced genomes

The obtained genomes presented a very consistent length, with the 2K-1 strain being superior in length compared to the other genomes, with approximately 6 Mbp in size. Most of the other strains had a length of 5.6 Mbp, with C3ACET53a and C1BHIC5 being larger by 0.2 Mbp. The GC content did not vary significantly, with most strains having approximately 66% GC content. The largest N50 value was observed for C1BHIC5 ( Table 2).

**Table 2.** Sequenced genomes described by length, Guanine + Cytosine percentage, and the N50 index.

Strain	Length (bp)	GC (%)	N50
2K-11	6489041	66.28	309354
3K-6	5661317	66.34	336908
6K11	5630190	66.29	336645
PB25	5661317	66.34	336908
C3ACET53a	5885892	66.46	376190
C1BHIC5	5845363	66.46	663224

Elaboration: Table created by the authors as part of this study.

### Average Nucleotide Indentity

**Figure 2.**
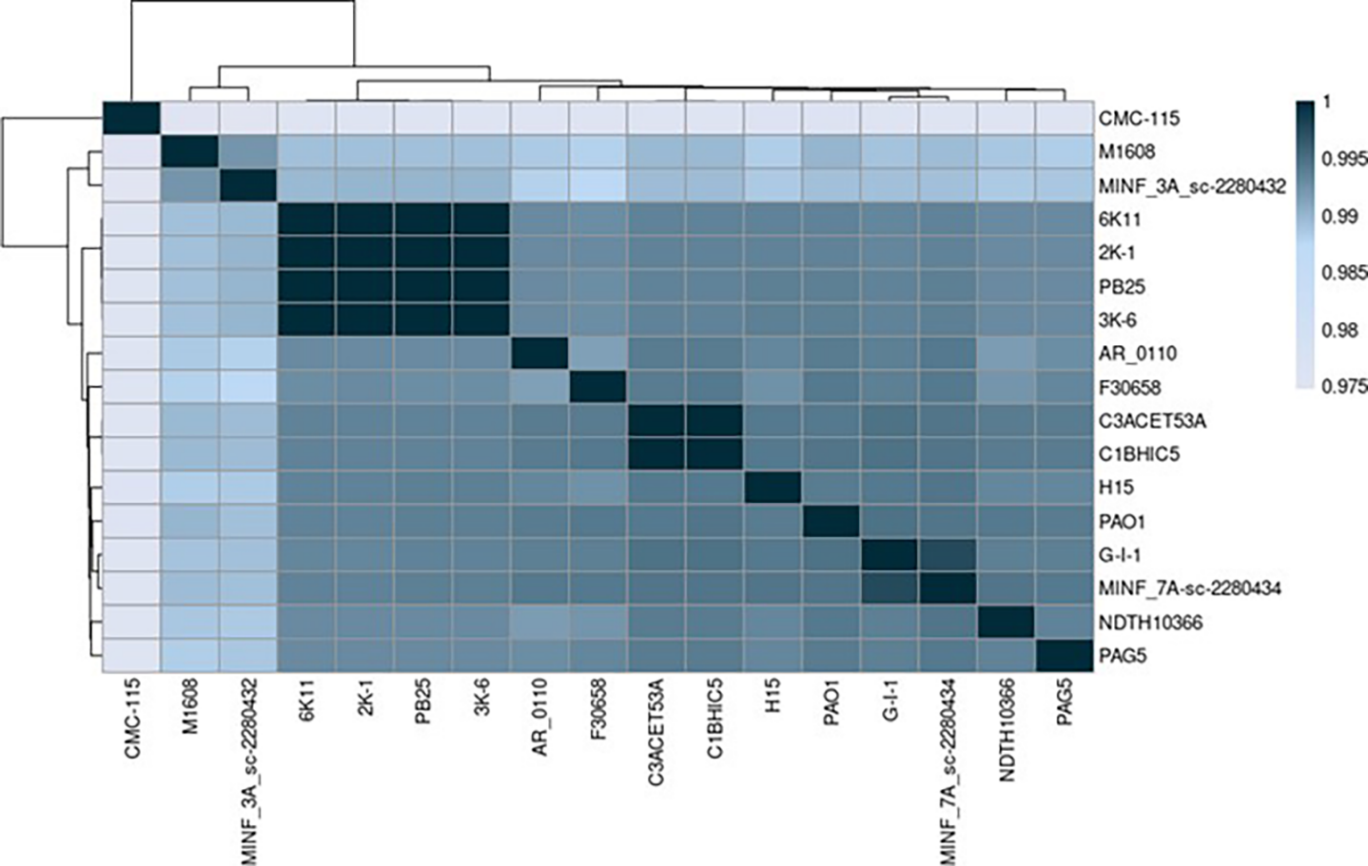
ANI heat map showing the ANI score between different strains in *Pseudomonas aeruginosa.* Elaboration: Figure created by the authors as part of this study.

The Average Nucleotide Identity (ANI) was compared between various strains of *Pseudomonas aeruginosa* using the algorithm MUMMER. The sequenced strains presented a notable similarity when compared to other *Pseudomonas aeruginosa* strains, verifying that our strains belong to the *Pseudomonas aeruginosa* species (>0.95) ( Jain et al., 2018). The lowest identity score was 97.5% (0.975), while the highest score was 99.9% (0.999) ( Figure 2).

### Pangenome

**Figure 3.**
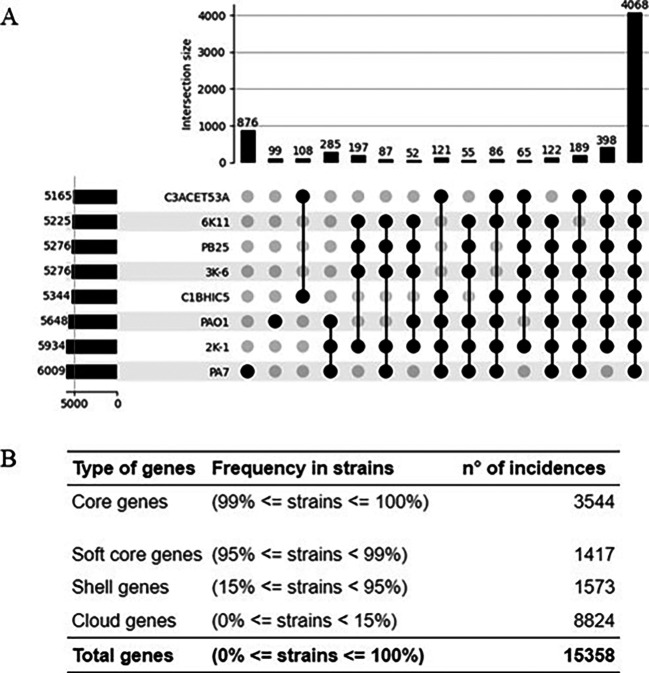
Pangenomic inference for the presented *Pseudomonas aeruginosa* strains. Elaboration: Figure created by the authors as part of this study.

A small pangenome is represented in Figure 3A, showing a comparison between the isolated strains, PAO1, and PA7. This comparison illustrates how the strains described in this work diverge from some representative strains of *Pseudomonas aeruginosa.* Among these 8 strains, 4068 genes are shared. Of these, 65 genes are common to all isolated strains, excluding PA7 and PAO1. Additionally, 108 genes are exclusively shared by C3ACET53A and C1BHIC5, while 197 genes are exclusively shared by the group comprising 6K-11, PB25, 3K-6, and 2K-1. This indicates certain similarities and shared genes among the isolates of interest, as well as a clear separation between the isolated strains into two distinct groups.

A total of 3544 genes were classified as core genes in a pangenome built with 93 strain, including the ones isolated for this study. These core genes are likely to be involved in essential cellular functions and housekeeping processes. On the other hand, 11814 genes were categorized as accessory or variable genes, reflecting their presence in only a subset of the genomes. These genes were classified in 3 categories: Softcore, shell and cloud genes, depending on their presence in the pangenome. These accessory genes contribute to the unique genetic diversity of each strain and may play crucial roles in shaping their distinct phenotypic characteristics ( Table 3).

**Table 3.** Comparison of *rhl* genes organization and synteny in different bacterial strains.

Characteristic	*Strain C1BHIC5*	*Strain C3ACET53A*	*Strain PAO1*	Other *strains*
Copy of *rhlB* gene	1 copy	2 copies	2 copies	2 copies
Position of *rhlABRI* gene	Adjacent to each other	Adjacent oriented 5′-3′	Adjacent oriented 3′-5′	Adjacent, oriented 3′-5′
Position of *rhlG* gene	Adjacent to *rhlC*	Adjacent to *rhlC, * oriented 3′-5′	Adjacent to *rhlC, * oriented 5′-3′	Adjacent to *rhlC*, oriented 5′-3′
Position of *rhlC* gene	Adjacent to *rhlG*	Adjacent to *rhlG*	Adjacent to *rhlG*	Adjacent to *rhlG*
Number of positions of *rhlB* gene	1 (within *rhlABRI* cluster)	2 (1 in *rhlABRI cluster, 1, elsewhere)*	2 (1 in *rhlABRI cluster, 1, elsewhere)*	2 (1 in *rhlABRI cluster, 1, elsewhere)*
Synteny of distant *rhlB*	Not applicable	Clear synteny	Clear synteny	Clear synteny

Elaboration: Table created by the authors as part of this study.

### Phylogeny “biotypes” found by Palleroni et al. (1973)

**Figure 4.**
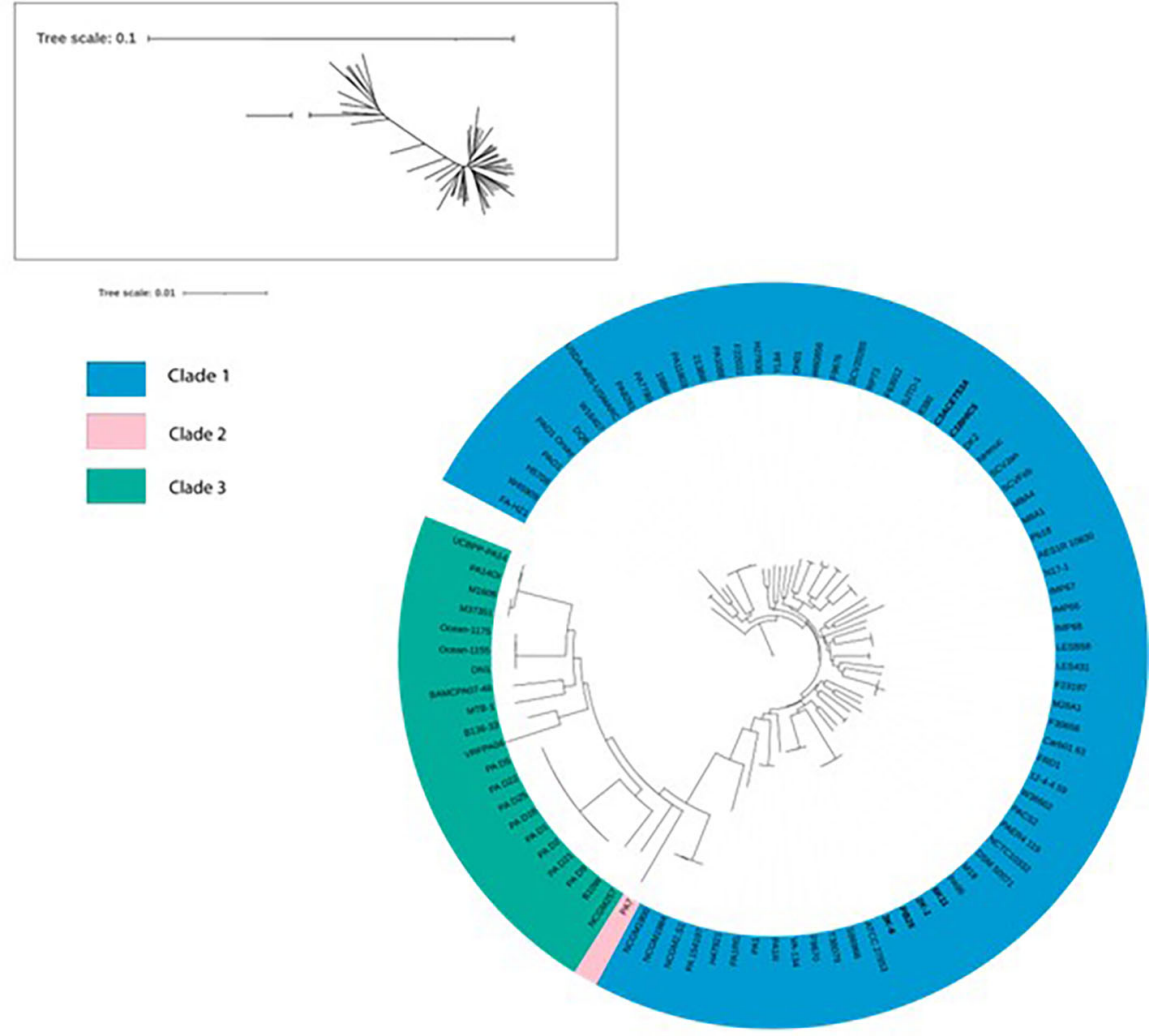
Phylogenetic tree using Maximum-Likelihood as provided by IQ-TREE. In bold letters, the isolates of interest. Elaboration: Figure created by the authors as part of this study.

A phylogenetic tree was constructed based on various strains previously reported in other studies. Bootstrap values reached a value of 100 for most cases. The branch length for PA7 had to be reduced because of its unusual divergence (original branch length = 9.9999513099). We attempted to reconstruct the *Pseudomonas aeruginosa* “biotypes” found by Palleroni et al. (1973). We determined that all six sequenced strains belong to Clade 1, in which there are also strains such as the reference strain PAO1. However, these strains are divided in 2 groups that are distant from each other in Clade 1. The 6K11, 2K-1, PB25 and 3K-6 strains are situated in a clade shared with strains T38079, F9670, S86968, ATCC 27853, PA96 and M18. Meanwhile, strains C1BHIC5 and C3ACET53A share a clade with strains 8380 and SJTD-1 ( Figure 4).

### Genomic comparative analysis

The *rhl* gene family, primarily found in *Pseudomonas aeruginosa* and *Burkholderia* species, is responsible for the biosynthesis of rhamnolipids. Rhamnolipids are surface-active molecules composed of rhamnose and *β*-hydroxydecanoic acid. These biosurfactants have been thoroughly investigated due to their involvement in various multicellular behaviors.

We evaluated the organization and synteny of *rhl* genes in several bacterial strains ( Figure 6). Our strains presented a double copy of the *rhlB* gene, except for the C1BHIC5 strain, with only 1 *rhlB* gene. The *rhl* genes did not exhibit a radically different organization across all evaluated strains: The genes *rhlABRI* are always neighboring each other. They are positioned in a 3′-5′ sense, except in C3ACET53A. This sense is the one exhibited in the PAO1 strain too. The *rhlG* gene does not have the same position in all strains. It always appears neighboring the *rhlC* gene. All *rhlG* genes in the strains have a 5′-3′ orientation, except in C3ACET53A. The *rhlC* gene, likewise, appears in different positions, but always neighboring the *rhlG* gene. Its orientation varies between strains. The *rhlB* gene is present in 2 positions in the genome. One copy is part of the *rhlABRI* cluster, while the other is situated in another position, far from the cluster. The *rhlABRI* cluster is also part of the PAO1 genome.

Furthermore, we observed a significant synteny between the *rhlB* gene in the recovered isolates. The copy situated far from the *rhlABRI* presents a clear synteny between all the strains, and seems to closely relate between each of these strains ( Table 3).

Due to the toluene tolerance previously shown by the strains ( Querebalú García, 2020, data not available), data no published), the *mla* system was sought as a gene operon of interest. Genomically, this system was present with a similar structure in all the isolated *P. aeruginosa* strains ( Figure 7). Almost all the strains exhibited two copies of the *mlaA* gene, except for the strain C1BHIC5, which presented only 1 copy of this gene. The *mlaFEDC* cluster is present in all strains. On the other hand, the *mlaEFD* cluster is present in all strains except for the strain C3ACET53A. The orientation of these clusters varies among the sequenced strains. Regarding the synteny between these clusters, it is clear that there was high synteny between these strains, and the *mla* gene blocks were highly similar. Interestingly, when compared with *Pseudomonas putida*, this species presented the *mla* genes between the *murA* and *ppcD* genes, in a similar conformation to other *P. aeruginosa* strains. Notably, the gene responsible for toluene tolerance, *ttg2D*, was also found among these genes in *P. putida.*

**Figure 5.**
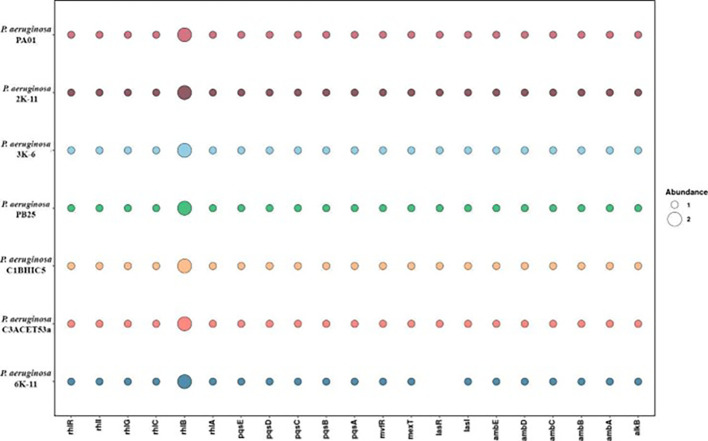
Presence absence of genes of interest for this study. Elaboration: Figure created by the authors as part of this study.

**Figure 6.**
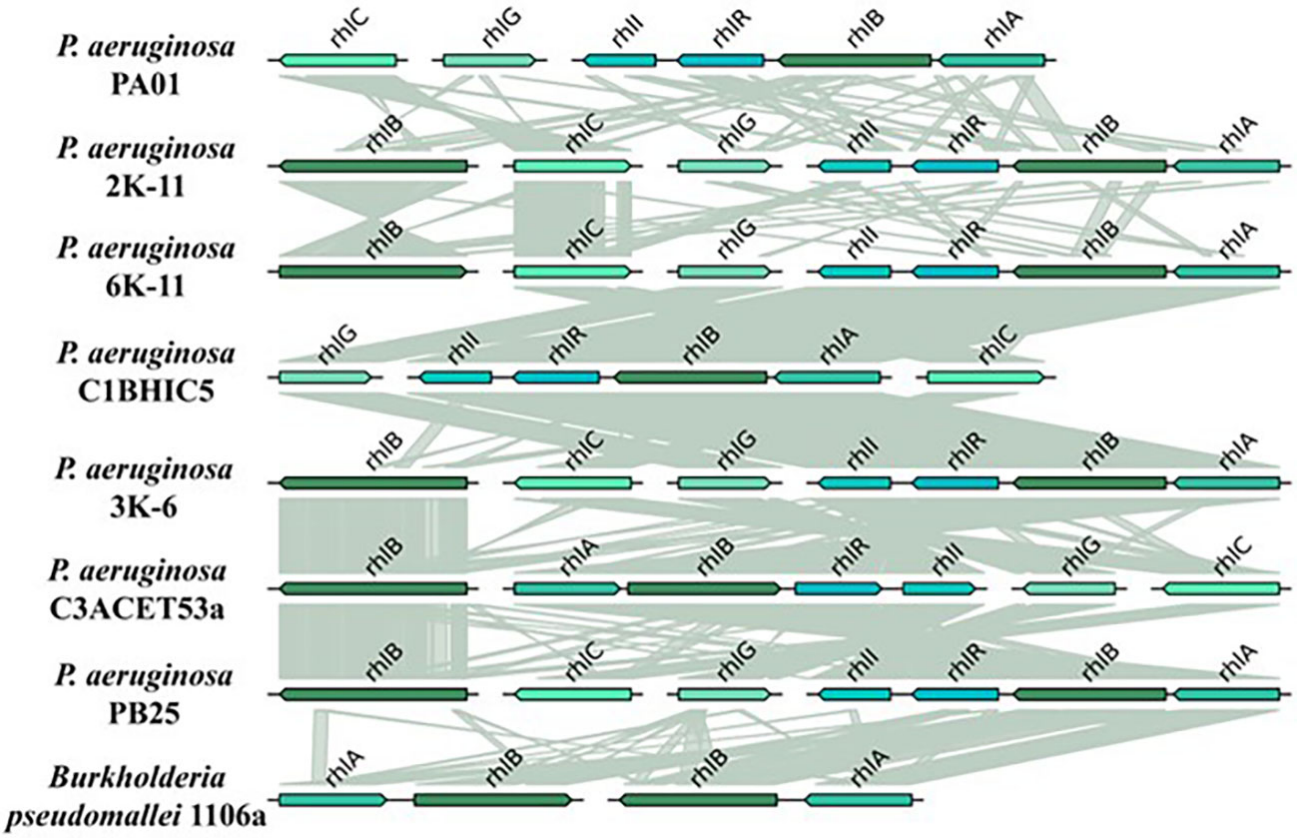
Gene topology aligned with tblastx for *rhl* system. Elaboration: Figure created by the authors as part of this study.

**Figure 7.**
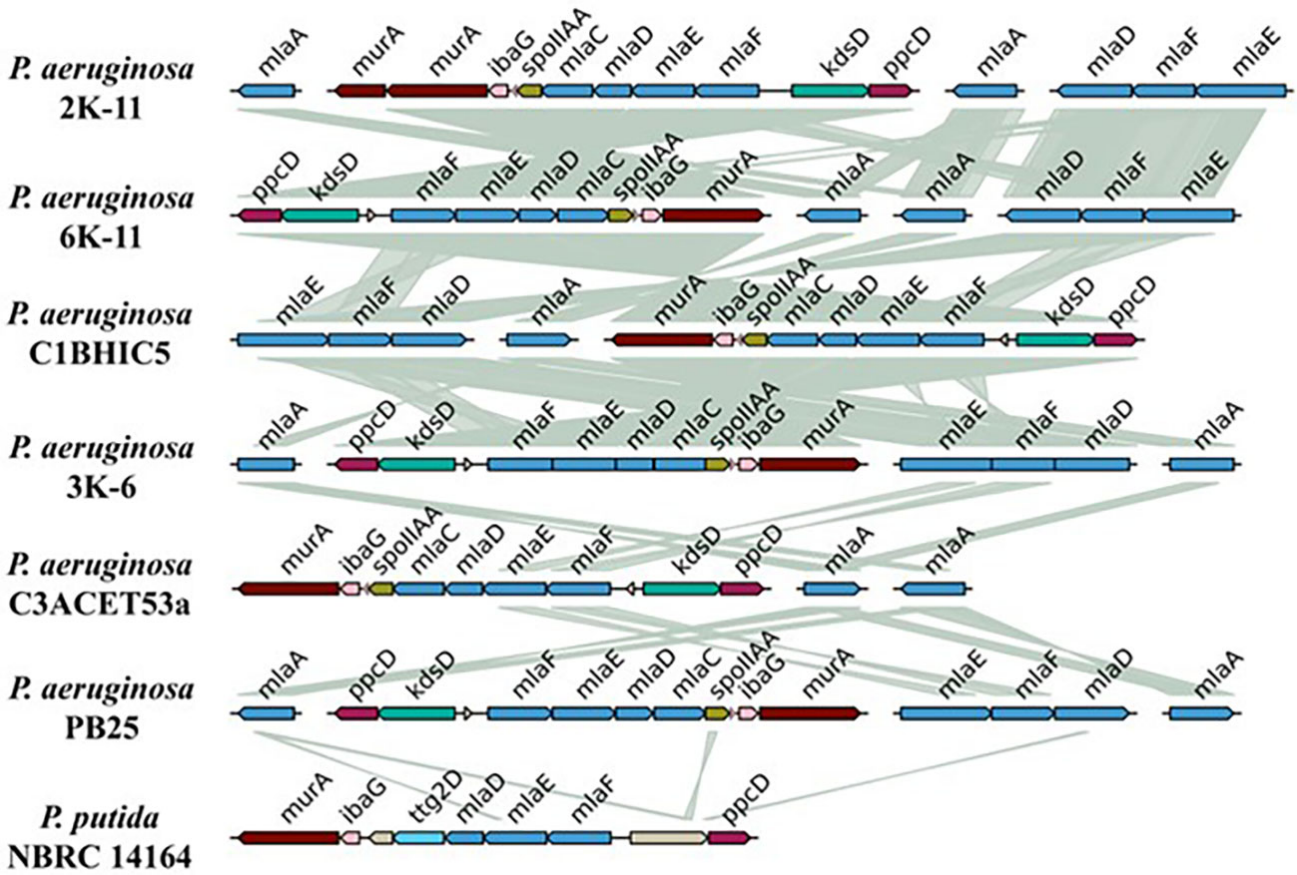
Gene topology aligned with tblastx for *mla* system. Elaboration: Figure created by the authors as part of this study.

## Discussion

### Strains isolated from Peruvian bioremediation effect

Several studies seek to evaluate the bioremediation potential of *Pseudomonas aeruginosa* against hydrocarbons, such as Baig et al. (2022), who isolated 2 strains: BAA-427 and ATCC-27853, from oil-contaminated soils (Aslam Refinery, Pakistan); Liu et al. (2022) also isolated *P. aeruginosa AQNU-1* in water samples from a lake wetland near a petrochemical industry (Anqing City, China) and Mahjoubi et al. (2021) isolated a halotolerant strain of *P. aeruginosa* UN14 in hydrocarbon-contaminated sediment (refinery on the coast of the port of Bizerte, northern Tunisia). In Peru, the Laboratory of Microbiology and Microbial Biotechnology of the U.N.M.S.M. has actively isolated strains of *Pseudomonas aeruginosa* from polluted environments, which present efficient physiological capacity in relation to hydrocarbon degradation. Of interest are strains with hyper-producing capacity of rhamnolipids ( *Pseudomonas aeruginosa* 6K-11); with emulsifying capacity and efficiency for the removal of heavy metals ( *Pseudomonas aeruginosa* PB25) and with hydrocarbonoclastic activity ( *Pseudomonas aeruginosa* C1BHIC5 and *Pseudomonas aeruginosa* C3ACETC53a).

### The new strains form 2 groups belonging to the Clade 1

The species phylogeny was constructed using strains previously reported in various studies, focusing especially on the “biotypes” identified by Palleroni et al. (1973). Our strains, originated from environmental sources, formed 2 distinct groups within Clade 1, along with clinical origin strains. Unlike other pathogenic bacteria, genomic studies have shown that clinical and environmental isolates of *P. aeruginosa* do not exhibit particular genomic differences, confirming that genetic variability related to the environment is reduced in this species ( Grosso-Becerra et al., 2014). This would explain why the origin of the strains did not determine the clade they formed in the phylogeny of *P. aeruginosa.* Likewise, it would also explain the results observed in the ANI matrix, in which the percentages are not very different from each other.

The pangenomic comparison also showed a large number of accessory genes (n=10,397; 67%) and a reduced core genome (n=3,544; 23%). This coincides with previous pangenomic studies that have highlighted the importance of horizontal gene transfer in this species ( Freschi et al., 2019).

### There is a set of genes related hydrocarbons degradation

*Pseudomonas aeruginosa* utilizes a quorum sensing (QS) system to regulate the synthesis of rhamnolipids (RLs), which are essential for the emulsification and assimilation of hydrocarbons ( Xu et al., 2020). The *rhlABC* genes are responsible for the production of these RLs in *Pseudomonas aeruginosa* strains isolated from petroleum-contaminated environments ( Castro et al., 2022; Ji et al., 2016). These genes encode the enzymes *RhlA*, *RhlB*, and *RhlC*, which transform precursors into mono-rhamnolipid and di-rhamnolipid. We have evidenced the presence of these genes in all strains analyzed through comparative genomic studies ( Figure 5).

Regarding quorum sensing interactions, the *las* system regulates the expression of virulence genes and activates the *pqs* and *rhl* systems ( Lee & Zhang, 2015), while the *pqs* system regulates more than 35 loci related to virulence ( Li et al., 2022). The *rhl* system controls both virulence genes and rhamnolipid production ( García-Reyes et al., 2020). In our analysis of these genes across several strains, we found that all strains have a similar gene abundance. However, the 6K-11 strain stands out due to the absence of the *lasR* gene ( Figure 7). These findings regarding the absence or presence of these genes could be due to the respective virulence mechanisms in each strain ( Dötsch et al., 2015).

In terms of the degradation of BTEX hydrocarbons (benzene, toluene, ethylbenzene, and xylene), specific genes responsible for this process have already been identified in various microorganisms ( Choi et al., 2013; Lee et al., 2019; Lin et al., 2002), primarily studied in species of *Pseudomonas* and *P. putida* ( Bacosa et al., 2021). In our study, in an attempt to corroborate hydrocarbonoclastic physiological activity, we searched for these genes within the complete genome but were unsuccessful in all the strains analyzed. Therefore, we searched for orthologous genes from the *ttg2* operon previously found in *Pseudomonas putida*, initially linked to toluene tolerance ( Yero et al., 2021), with this system being the *mla.* *MlaA* removes glycerophospholipids from the outer leaflet of the outer membrane (OM) and deliver them to the *MlaFEDB* complex in the inner membrane (IM) via the periplasmic substrate-binding protein *MlaC* ( Kaur et al., 2023).

The genetic organization of the *mla* system in the synteny showed discontinuity, as the *mlaCDEF* genes are grouped in an operon, while *mlaA* is separated in the genome. This aligns with what was reported by Kaur and Mingeot -Leclercq (2024), who mentioned that in gram-negative bacteria, including *P. aeruginosa*, the system is organized in this way. However, the *mlaB* gene was not found in the operon, consistent with the absence of this gene in alpha-proteobacteria and epsilon-proteobacteria.

Benzene, toluene, ethylbenzene, and xylene, collectively known as BTEX, are common pollutants of soils and groundwater. BTEX and other polycyclic aromatic hydrocarbons are extremely toxic to microorganisms due to their accumulation in hydrophobic cell membranes ( Choi et al., 2013; Hąc-Wydro et al., 2019). Therefore, the genes studied may be involved in resistance to BTEX. *Pseudomonas aeruginosa* possesses a complex genetic and enzymatic regulatory system that allows it not only to produce rhamnolipids essential for the emulsification and assimilation of hydrocarbons but also to tolerate aromatic compounds such as BTEX

## Conclusions

Studies on genes related to the degradation of monoaromatic hydrocarbons have been conducted in *Pseudomonas putida.* The toluene-degrading plasmid (TOL) has been studied in *P. putida* mt-2, which encodes metabolic pathways for the degradation of toluene, *m*-xylene, and *p*-xylene into carboxylic acids. The *xylN* genes of the TOL plasmid encode a porin transporter for *m*-xylene ( Kasai et al., 2001). Similarly, toluene degradation follows six differentiated metabolic pathways, involving the *tod* gene, which encodes the enzyme toluene dioxygenase, the *tmo*/ *tbm*/ *tbc* genes responsible for the synthesis of the Toluene 2-monooxygenase enzyme, *tbu*/ *tbh* genes that encode Toluene 3-monooxygenase, *tmoABCDEF* genes that encode different subunits of the ortho Toluene 4-monooxygenase protein, and *tbc1FEDCBA*/ *tbc2ABCDEF* genes that encode meta Toluene 4-monooxygenase ( Parales et al., 2008). On the other hand, the genes involved in the aerobic degradation of benzene are the *benzA* genes, which encode an enzyme that carries out the initial oxidation reaction of the ring-cleavage of monoaromatic hydrocarbons, and this enzyme has been studied in *Pseudomonas putida* AQ8 ( Chicca et al., 2020). To date, there are not many records involving strains of the species *Pseudomonas aeruginosa* in the degradation of monoaromatic hydrocarbons, nor annotated genes associated with this process.

## Submitting your article

If you are using Overleaf, either select “Submit” then F1000Research, or click “Submit to F1000Research” in the top right-hand corner. Alternatively, generate a PDF file of your project and submit this alongside a zip file containing all project files (includes the source files, style files, and PDF) using online submission form.

## Ethics and consent

'Ethical approval and consent were not required.

## Data Availability

All genomes have been uploaded to the NCBI repository. Code used for this study is stored in the following GitHub repository: https://github.com/reymonerapseudomonas_notebook.git GitHub: A comparative genomic analysis of Pseudomonas aeruginosa strains isolated from oil-contaminates environments in Peru (Script Report). https://doi.org/10.5281/zenodo.14210953; ( Castillo-Vilcahuaman, 2024a) Data is available under the terms of the Creative Commons Zero “No rights reserved” data waiver (CC0 1.0 Public domain dedication). Zenodo: Comparative genomics of genes for rhamnolipid synthesis and monoaromatic hydrocarbon tolerance in environmental strains of *Pseudomonas aeruginosa, Doi:* https://doi.org/10.5281/zenodo.14210953; (From this paper) ( Castillo-Vilcahuaman, 2024a) The project contains the following underlying data:
• reymonera/pseudomonas_notebook-v.1.0.zip Data are available under the terms of the Creative Commons Attribution 4.0 International license (CC-BY 4.0). Accession number: These assemblies have been deposited at DDBJ/ENA/GenBank under the accessions: GenBank: Assembled Genome for *Pseudomonas aeruginosa* strain 2K-1. Accession number GCA_024706435.1; https://www.ncbi.nlm.nih.gov/datasets/genome/GCA_024706435.1/; (From this paper) GenBank: Assembled Genome for *Pseudomonas aeruginosa* strain 6K-11 Accession number GCA_025380225.1; https://www.ncbi.nlm.nih.gov/datasets/genome/GCA_025380225.1/; (From this paper) GenBank: Assembled Genome for *Pseudomonas aeruginosa* strain C1BHIC5. Accession number GCA_025210085.1; https://www.ncbi.nlm.nih.gov/datasets/genome/GCA_025210085.1/; (From this paper) GenBank: Assembled Genome for *Pseudomonas aeruginosa* strain 3K-6 Accession number GCA_025209925.1; https://www.ncbi.nlm.nih.gov/datasets/genome/GCA_025209925.1/; (From this paper) GenBank: Assembled Genome for *Pseudomonas aeruginosa* strain PB25 Accession number GCA_025209735.1; https://www.ncbi.nlm.nih.gov/datasets/genome/GCA_025209735.1/; (From this paper) GenBank: Assembled Genome for *Pseudomonas aeruginosa* strain C3ACETC53a Accession number GCA_025210135.1; https://www.ncbi.nlm.nih.gov/datasets/genome/GCA_025210135.1/; (From this paper)

## Supplementary data

Figshare: ANIm Percentage Identity Tabular Data. AN MatrixI: https://doi.org/10.6084/m9.figshare.27895758.v1 ( Castillo-Vilcahuaman, 2024b).

Figshare: Pangenome Presence-Absence Tables, https://doi.org/10.6084/m9.figshare.27895629.v1 ( Castillo-Vilcahuaman, 2024c).

The project contains the following extended data:

• ANIm_percentage_identity.tab
• collapsed_gene_presence_absence.csv
• gene_presence_absence.csv

Data are available under the terms of the Creative Commons Zero license.
