# Supplementary material for: Comparative Genomics of Rhamnolipid Synthesis and Monoaromatic Hydrocarbon Tolerance Genes in Environmental Pseudomonas aeruginosa strains
Source: F1000Res. 2025 Apr 17;13:1519. Originally published 2024 Dec 13. [Version 2] doi: 10.12688/f1000research.158761.2 (PMC12035672; doi:10.12688/f1000research.158761.2)
Supplement: Peer review history

# Reviewer report: Reviewer response for version 2

**DOI:** 10.5256/f1000research.179795.r378707

**Published:** 2025-04-24

**Authors:** Andi Kurniawan

**Affiliations:** 1 University of Brawijaya, Malang, Indonesia

I do not have further comments.

Is the work clearly and accurately presented and does it cite the current literature?

Yes

If applicable, is the statistical analysis and its interpretation appropriate?

Partly

Are all the source data underlying the results available to ensure full reproducibility?

Yes

Is the study design appropriate and is the work technically sound?

Partly

Are the conclusions drawn adequately supported by the results?

Partly

Are sufficient details of methods and analysis provided to allow replication by others?

Partly

Reviewer Expertise:

Microbial ecology, biofilms, environmental biotechnology, aquatic resources

I confirm that I have read this submission and believe that I have an appropriate level of expertise to confirm that it is of an acceptable scientific standard.

# Reviewer report: Reviewer response for version 1

**DOI:** 10.5256/f1000research.174398.r353950

**Published:** 2025-03-03

**Authors:** Wijdan H Al-tamimi

**Affiliations:** 1 University of Basrah, Basrah, Iraq

The research demonstrates remarkable scientific quality through its integrated methodology and use of modern genomic analysis tools. The use of comparative analyses such as the calculation of average nucleotide identity (ANI) and the construction of an evolutionary tree using tools such as iTOL enhances the reliability of the results and provides a clear view of the genetic diversity between the strains.

 It is also positive that the study compared the rhamnolipid production gene system (rhl) and the BTEX tolerance system (mla) in the context of biodegradation capabilities of pollutants, a topic of great environmental importance. The provision of genomic data and transparency in presenting the sources and software used are strengths that contribute to the replication of the experiment and the verification of the results. However, the research may be criticized in terms of the relative number of samples (six strains), which may limit the possibility of generalizing the results on a wider scale. There are also some points that may need additional clarification or functional experimental support to strengthen the link between genetic structure and biological functions.

Is the work clearly and accurately presented and does it cite the current literature?

Yes

If applicable, is the statistical analysis and its interpretation appropriate?

Yes

Are all the source data underlying the results available to ensure full reproducibility?

Yes

Is the study design appropriate and is the work technically sound?

Yes

Are the conclusions drawn adequately supported by the results?

Yes

Are sufficient details of methods and analysis provided to allow replication by others?

Yes

Reviewer Expertise:

Applied Microbiology

I confirm that I have read this submission and believe that I have an appropriate level of expertise to confirm that it is of an acceptable scientific standard.

## References

[1] : Enhancing the spontaneous imbibition process using biosurfactants produced from bacteria isolated from Al-Rafidiya oil field for improved oil recovery. *Journal of Petroleum Exploration and Production Technology* .2020;10(8) : 10.1007/s13202-020-00874-9 3767-3777 10.1007/s13202-020-00874-9

[2] : Distribution and molecular characterization of biosurfactant-producing bacteria. *Biodiversitas Journal of Biological Diversity* .2020;21(9) : 10.13057/biodiv/d210914 10.13057/biodiv/d210914

# Reviewer report: Reviewer response for version 1

**DOI:** 10.5256/f1000research.174398.r351909

**Published:** 2025-02-12

**Authors:** Andi Kurniawan

**Affiliations:** 1 University of Brawijaya, Malang, Indonesia

**Review Report: "Comparative Genomics of Genes for Rhamnolipid Synthesis and Monoaromatic Hydrocarbon Tolerance in Environmental Strains of Pseudomonas aeruginosa"**

**General Comments:**

 The manuscript investigates the comparative genomics of genes associated with rhamnolipid synthesis and monoaromatic hydrocarbon tolerance in six Pseudomonas aeruginosa strains isolated from hydrocarbon-polluted environments in Peru. While the study addresses a pertinent topic in environmental microbiology and offers potential insights into bioremediation strategies, several critical issues need to be addressed to enhance the clarity, rigor, and impact of the manuscript.

**Comments and Revision Suggestions:**

***Abstract***

 The abstract does not clearly articulate its significance. Acronyms like BTEX are introduced without explanation, potentially confusing readers. Furthermore, the methodology and main findings are not summarized concisely, and the conclusion lacks a clear statement of the study's practical implications.

- Define BTEX (benzene, toluene, ethylbenzene, and xylene) upon first mention.
- Explicitly state the research gap and highlight the significance of studying these genes in Pseudomonas aeruginosa.
- Briefly outline the key methods used, such as genome sequencing and comparative analysis, to provide readers with an understanding of the approach.
- Summarize the major findings concisely and emphasize their implications for bioremediation.

***Introduction***

 The introduction lacks a clear articulation of the research gap and the study's specific objectives. The background on the mla system and its role in hydrocarbon degradation is insufficient, and the rationale for selecting strains from Peru is unclear.

- Provide a more comprehensive background on BTEX pollutants and their environmental impact.
- Expand on the mla system’s relevance to BTEX tolerance and biodegradation in Pseudomonas aeruginosa.
- Clearly state the research question or hypothesis at the end of the introduction.
- Justify the focus on Peruvian strains, emphasizing any unique environmental factors or characteristics.

***Methods***

 The methodology lacks detail, making replication challenging. Parameters for tools and thresholds are not consistently reported, and the criteria for selecting reference genomes are unclear.

- Provide detailed protocols for DNA extraction, sequencing (e.g., platform, read length, and coverage), and quality control.
- Include software versions and specify parameters used for bioinformatics tools (e.g., ANI thresholds, synteny criteria).
- Clarify the selection criteria for reference genomes and justify their inclusion in the comparative analysis.
- Describe the statistical methods used for analyzing data, ensuring transparency and reproducibility.

***Results***

 The results section often blends data presentation with interpretation, which should be reserved for the discussion. Figures and tables are insufficiently annotated, and the significance of certain findings (e.g., variations in gene organization) is not fully explored.

- Separate data presentation from its interpretation, reserving the latter for the discussion section.
- Enhance figure and table captions to include all necessary details for understanding without referring to the main text.
- Provide a deeper analysis of genetic variations, particularly in the rhl and mla gene clusters, and their potential impact on hydrocarbon degradation.
- Discuss the ecological implications of the observed accessory genes in the pangenome analysis.

***Discussion***

 The discussion fails to critically analyze the findings or situate them within a broader scientific context. Potential applications of the findings in bioremediation are not adequately addressed, and some claims lack sufficient evidence.

- Deepen the analysis of gene organization and its impact on rhamnolipid synthesis and hydrocarbon tolerance.
- Compare the findings with previous studies on Pseudomonas aeruginosa and related species, highlighting similarities and differences.
- Discuss practical applications, such as the potential use of these strains in bioremediation or synthetic biology.
- Ensure all claims are substantiated with data or appropriate references to existing literature.

***Conclusion***

 The conclusion is overly brief and does not effectively summarize the study's contributions or implications.

- Provide a concise summary of the key findings and their significance.
- Highlight how the study advances knowledge in microbial genomics and hydrocarbon bioremediation.
- Suggest future research directions based on the results, such as further exploration of gene-environment interactions.

***Figures and Tables***

 Some figures are of low resolution, lack essential annotations, or have insufficient captions. Tables contain unexplained abbreviations and inconsistent formatting.

- Ensure all figures are of high resolution, with clear labels and comprehensive legends.
- Expand figure captions to include detailed explanations of data, symbols, and abbreviations.
- Review all tables for consistency and clarity, providing explanations for abbreviations where necessary.

***References***

 The references contain inconsistencies in formatting, and some key studies relevant to the research are missing.

- Ensure all references are formatted according to the journal's guidelines and maintain consistency throughout.
- Include additional recent studies to strengthen the discussion, particularly those focusing on BTEX degradation and Pseudomonas aeruginosa genomics.
- Cross-check in-text citations with the reference list to ensure accuracy and completeness.

Is the work clearly and accurately presented and does it cite the current literature?

Yes

If applicable, is the statistical analysis and its interpretation appropriate?

Partly

Are all the source data underlying the results available to ensure full reproducibility?

Yes

Is the study design appropriate and is the work technically sound?

Partly

Are the conclusions drawn adequately supported by the results?

Partly

Are sufficient details of methods and analysis provided to allow replication by others?

Partly

Reviewer Expertise:

Microbial ecology, biofilms, environmental biotechnology, aquatic resources

I confirm that I have read this submission and believe that I have an appropriate level of expertise to confirm that it is of an acceptable scientific standard, however I have significant reservations, as outlined above.

# Response

**Published:** 2025-03-23

**Authors:** ROGER ALBERTO PALOMINO HUARCAYA

- **Abstract: **The abstract was revised to define BTEX (benzene, toluene, ethylbenzene, and xylene) explicitly upon first mention. Additionally, it now better highlights the significance of studying these genes in Pseudomonas aeruginosa and their potential implications for bioremediation. A concise summary of the methodology was included, and the main findings were emphasized with a clearer statement of their relevance.
- **Introduction: **The introduction was refined to provide a stronger background on BTEX pollutants and their environmental impact. Regarding the mla system, the revision acknowledges that while there are no definitive studies confirming its direct role in BTEX tolerance, it could potentially contribute to stress resistance by maintaining membrane asymmetry. This possibility was discussed in the manuscript, presenting it as a hypothesis rather than a confirmed function. Additionally, the rationale for focusing on Peruvian strains was strengthened by emphasizing potential environmental factors that make these isolates relevant for studying hydrocarbon degradation.
- **Methods: **The methodology was expanded to improve reproducibility. All software versions were explicitly reported and properly cited, and additional details on bioinformatics analyses were provided. Parameters for tools and selection criteria for reference genomes were clarified to ensure transparency.
- **Results: T**he section was refined to focus strictly on data presentation. Any interpretative content that previously blended discussion with results was removed and relocated to the Discussion section.
- **Discussion: **This section was expanded to include a more in-depth comparative analysis with previous studies on Pseudomonas aeruginosa and related species. The potential applications of the findings in bioremediation and synthetic biology were discussed in greater depth. However, the role of accessory genes was not further explored, as it is outside the primary scope of this study.
- **Conclusion: **The conclusion was revised to provide a clearer summary of the study’s key findings and their significance. Future research directions were also suggested, particularly regarding further investigations into gene-environment interactions and functional studies to validate the role of the studied gene clusters in hydrocarbon degradation.
- **Figures and Tables:** Figures and tables were improved with clearer labels and more descriptive captions. High-resolution versions of the figures were submitted to enhance their quality for publication.
